# Correction: Does a rise in BMI cause an increased risk of diabetes?: Evidence from India

**DOI:** 10.1371/journal.pone.0247537

**Published:** 2021-02-19

**Authors:** Shivani Gupta, Sangeeta Bansal

There are errors in the Funding statement. The correct Funding statement is as follows: This study was partially supported by Jawaharlal Nehru University in providing financial support towards the publication fee for this article. No additional external funding was received for this study.
