# Supplementary material for: Does a rise in BMI cause an increased risk of diabetes?: Evidence from India
Source: PLoS One. 2020 Apr 1;15(4):e0229716. doi: 10.1371/journal.pone.0229716 (PMC7112218; doi:10.1371/journal.pone.0229716)
Supplement: S1 Appendix — (DOCX) [file pone.0229716.s001.docx]

**S1 Appendix: Statistical Analytical Codes used in the Analysis**

// STATISTICAL ANALYTICAL CODE for the paper titled "Does a rise in BMI causes an increased risk of diabetes?: Evidence from India"//

// By Shivani Gupta1¶ and Sangeeta Bansal1¶*//

//1 Centre for International Trade and Development, School of International Studies, Jawaharlal Nehru University, New Delhi, India.//

//* Corresponding author//

//¶ These authors contributed equally to this work**//**

//E-mail: sangeeta.bansal7@gmail.com//

// *****Regrssions estimated using STATA SOFTWARE***** //

************LIST OF VARIABLES ALONG WITH DEFINITIONS*****************

(1) diabetesdummy_mf_both = Self-Reported Diabetes Status

• D = 0 if non-diabetic

• D = 1 if diabetic

(2) bloodglucose_ord = Ordinal Blood Glucose Levels

• BG = 0 if blood glucose is less than or equal to140 mg/dl

• BG = 1 if between 141 and 200 mg/dl

• BG = 2 if higher than 200 mg/dl

(3) bmi = Body Mass Index - Person’s weight is kilograms divided by square of his/her height in meters (kg/m2).

(4) bmi_head = Body Mass Index of the head of the family for the married couples sub-sample

(5) bmi_wife_husb = Body Mass Index of the spouse of the head of the family for the married couples sub-sample

(6) age = Age of the individual (in years).

(7) female = Gender dummy

• = 0 if Male

• = 1 if Female

(8) bankaccnt = Has Bank Account

• = 0 if individual does not have bank account

• = 1 if individual has bank account

(9) wealth = Wealth Quintile

• = 0 if poorest

• = 1 if poorer

• = 2 if middle

• = 3 if richer

• = 4 if richest

(10) religion = Religion

• = 0 if Hindu

• = 1 if Muslim

• = 2 if Christian

• = 3 if Sikh

• = 4 if Buddhist/neo-Buddhist

• = 5 if Jain

• = 6 if Jewish

• = 7 if Parsi/Zoroastrian

• = 8 if no religion

• = 9 if some other religion

(11) sc = Shedueld Caste

• = 1 if Scheduled Caste, 0 otherwise

(12) st = Scheduled Tribe

• = 1 if Scheduled Tribe, 0 otherwise

(13) obc = Other Backward Classes

• = 1 if Other Backward Classes, 0 otherwise

(14) insurance = Insurance

• = 0 if any usual member of household is not covered by a health scheme or health insurance

• = 1 if any usual member of household is covered by a health scheme or health insurance

(15) bpl = Below Poverty Line

• = 0 if household does not have BPL card

• = 1 if household has BPL card

(16) familystruc = Family Structure

• = 0 if nuclear family

• = 1 if non-nuclear or joint family

(17) education = Educational attainment

• = 0 if no education or preschool

• = 1 if Primary

• = 2 if Secondary

• = 3 if Higher

(18) hh_members = Number of Household Members - Number of total household members in all age groups.

(19) region = Region

• = 0 if Rural

• = 1 if Urban

(20) last_ate = Time since last ate - Time since last ate (in hours). Time is recorded before blood glucose measurements are taken.

(21) last_drink = Time since last drink - Time since last drink (in hours), something other than plain water. Time is recorded before blood glucose measurements are taken.

(22) smokecigrtt = 1 if smokes cigarette, 0 otherwise

(23) smokepipe = 1 if smokes pipe, 0 otherwise

(24) chewtobacco = 1 if chews tobacco, 0 otherwise

(25) snuff = 1 if snuffs, 0 otherwise

(26) smokecigar = 1 if smokes cigar, 0 otherwise

(27) paangutkha = 1 if chews paan or gutkha, 0 otherwise

(28) paanwithtobacco = 1 if chews paan with tobacco, 0 otherwise

(29) drinkalcohol = 1 if drinks alcohol, 0 otherwise

(30) fried1 = 1 if eats fried food daily or weekly, 0 otherwise

(30) aerated1 = 1 if drinks aerated drink daily or weekly, 0 otherwise

(31) statecode = coding used for each state and union territory **takes value 1 to 36 - for 36 states and union territories of India**

(32) overweigh_obese = overweight and obesity status dummy (WHO criteria) - for full sample

• = 0 if BMI is less than 25 kg/m2

• = 1 if BMI is graeter than or equal to 25 kg/m2

(33) overweigh_obese23 = overweight and obesity status dummy (Asian criteria) - for full sample

• = 0 if BMI is less than 23 kg/m2

• = 1 if BMI is graeter than or equal to 23 kg/m2

(34) overobs25 = overweight and obesity status dummy (WHO criteria) - for full sample

• = 0 if BMI is less than 25 kg/m2

• = 1 if BMI is graeter than or equal to 25 kg/m2

(34) overobs23 = overweight and obesity status dummy (Asian criteria) - for full sample

• = 0 if BMI is less than 23 kg/m2

• = 1 if BMI is graeter than or equal to 23 kg/m2

(36) ovrdumbmihead25 = overweight and obesity status dummy (WHO criteria) - for married couples sub-sample

• = 0 if BMI is less than 25 kg/m2

• = 1 if BMI is graeter than or equal to 25 kg/m2

(37) ovrdumbmihead23 = overweight and obesity status dummy (Asian criteria) - for married couples sub-sample

• = 0 if BMI is less than 23 kg/m2

• = 1 if BMI is graeter than or equal to 23 kg/m2

(38) dumbmihwpositive = 1 if BMI of spouse available, = 0 otherwise

// Table 1

**Descriptive Statistics by Overweight or Obesity Status**

** Following command gives us mean values of the variables grouped by their overweight and obesity status**

** We obtain mean across two categories - Overweight or Obese and Non-Overweight, along with difference between the along with t-statistic values**

ttest varibale, by( overweigh_obese)

// Table 2

**Average Marginal Effects of BMI on Self-Reported Diabetes Status: Probit and IV-Probit Model Estimates for Married Couples Sub-Sample**

**Porbit Model**

**Columns 1 and 2**

probit diabetesdummy_mf_both bmi_head age i.female bankaccnt i.wealth i.religion sc st obc insurance bpl i.familystruc i.education hh_members region smokecigrtt smokepipe chewtobacco snuff smokecigar paangutkha paanwithtobacco drinkalcohol fried1 aerated1 i.statecode if dumbmihwpositive==1 , vce(robust)

margins, dydx ( bmi_head ) pred(pr) over ( ovrdumbmihead25) pwcompare (group)

margins, dydx ( bmi_head ) pred(pr) over ( ovrdumbmihead25) pwcompare

margins, dydx ( bmi_head ) pred(pr) over ( ovrdumbmihead23) pwcompare (group)

margins, dydx ( bmi_head ) pred(pr) over ( ovrdumbmihead23) pwcompare

**IV-Probit Model**

**Columns 3 and 4**

ivprobit diabetesdummy_mf_both age i.female bankaccnt i.wealth i.religion sc st obc insurance bpl i.familystruc i.education hh_members region smokecigrtt smokepipe chewtobacco snuff smokecigar paangutkha paanwithtobacco drinkalcohol fried1 aerated1 i.statecode (bmi_head = bmi_wife_husb) , vce(cluster statecode)

margins, dydx ( bmi_head ) pred(pr) over ( ovrdumbmihead25) pwcompare (group)

margins, dydx ( bmi_head ) pred(pr) over ( ovrdumbmihead25) pwcompare

margins, dydx ( bmi_head ) pred(pr) over ( ovrdumbmihead23) pwcompare (group)

margins, dydx ( bmi_head ) pred(pr) over ( ovrdumbmihead23) pwcompare

**First-Stage Regression of IV-Probit Model**

** F-statistic and R square**

reg bmi_head bmi_wife_husb age i.female bankaccnt i.wealth i.religion sc st obc insurance bpl i.familystruc i.education hh_members region smokecigrtt smokepipe chewtobacco snuff smokecigar paangutkha paanwithtobacco drinkalcohol fried1 aerated1 i.statecode

// Table 3

**Average Marginal Effects of BMI on Self-Reported Diabetes Status amongst Overweight or Obese Individuals (BMI = 25 kg/m2): Probit and IV-Probit Model Estimates for Married Couples Sub-Sample**

**Porbit Model**

**Columns 1, 2 and 3**

probit diabetesdummy_mf_both bmi_head age i.female bankaccnt i.wealth i.religion sc st obc insurance bpl i.familystruc i.education hh_members region smokecigrtt smokepipe chewtobacco snuff smokecigar paangutkha paanwithtobacco drinkalcohol fried1 aerated1 i.statecode if bmi_head>=25 & dumbmihwpositive==1 , vce(robust)

margins, dydx ( bmi_head ) pred(pr) over ( female_new)

margins, dydx ( bmi_head ) pred(pr) over ( female_new) pwcompare (group)

margins, dydx ( bmi_head ) pred(pr) over ( female_new) pwcompare

margins, dydx ( bmi_head ) pred(pr) over ( region)

margins, dydx ( bmi_head ) pred(pr) over ( region) pwcompare (group)

margins, dydx ( bmi_head ) pred(pr) over ( region) pwcompare

margins, dydx ( bmi_head ) pred(pr) over ( wealth)

margins, dydx ( bmi_head ) pred(pr) over ( wealth) pwcompare (group)

margins, dydx ( bmi_head ) pred(pr) over ( wealth) pwcompare

**IV-Porbit Model**

**Columns 4, 5 and 6**

ivprobit diabetesdummy_mf_both age i.female bankaccnt i.wealth i.religion sc st obc insurance bpl i.familystruc i.education hh_members region smokecigrtt smokepipe chewtobacco snuff smokecigar paangutkha paanwithtobacco drinkalcohol fried1 aerated1 i.statecode (bmi_head = bmi_wife_husb) if bmi_head>=25 , vce(cluster statecode)

margins, dydx ( bmi_head ) pred(pr) over ( female_new)

margins, dydx ( bmi_head ) pred(pr) over ( female_new) pwcompare (group)

margins, dydx ( bmi_head ) pred(pr) over ( female_new) pwcompare

margins, dydx ( bmi_head ) pred(pr) over ( region)

margins, dydx ( bmi_head ) pred(pr) over ( region) pwcompare (group)

margins, dydx ( bmi_head ) pred(pr) over ( region) pwcompare

margins, dydx ( bmi_head ) pred(pr) over ( wealth)

margins, dydx ( bmi_head ) pred(pr) over ( wealth) pwcompare (group)

margins, dydx ( bmi_head ) pred(pr) over ( wealth) pwcompare

// Table 4

**Average Marginal Effects of BMI on Ordinal Blood Glucose Levels: Ordered Probit Model Estimates based on Full Sample Data**

oprobit bloodglucose_ord bmi age i.female bankaccnt i.wealth i.religion sc st obc insurance bpl i.familystruc i.education married hh_members region last_ate last_drink smokecigrtt smokepipe chewtobacco snuff smokecigar paangutkha paanwithtobacco drinkalcohol fried1 aerated1 i.statecode , vce(robust)

margins , dydx( bmi ) over ( overobs25 ) pwcompare (group)

margins , dydx( bmi ) over ( overobs25 ) pwcompare

margins , dydx( bmi ) over ( overobs23 ) pwcompare (group)

margins , dydx( bmi ) over ( overobs23 ) pwcompare

// Table 5

**Average Marginal Effects of BMI on Ordinal Blood Glucose Levels amongst Overweight or Obese Individuals (BMI â‰¥ 25 kg/m2): Ordered Probit Model Estimates based on Full Sample Data**

oprobit bloodglucose_ord bmi age i.female bankaccnt i.wealth i.religion sc st obc insurance bpl i.familystruc i.education married hh_members region last_ate last_drink smokecigrtt smokepipe chewtobacco snuff smokecigar paangutkha paanwithtobacco drinkalcohol fried1 aerated1 i.statecode if bmi>=25, vce(robust)

margins , dydx( bmi ) over (female) pwcompare (group)

margins , dydx( bmi ) over (female) pwcompare

margins , dydx( bmi ) over (region) pwcompare (group)

margins , dydx( bmi ) over (region) pwcompare

margins , dydx( bmi ) over (wealth) pwcompare (group)

margins , dydx( bmi ) over (wealth) pwcompare

////**** Supplementary Information****////

// S1 Table

**List of Variables with Definition and Type**

** This table is provided in supplementary information - no statistical code needed**

// S2 Table

**Descriptive Statistics**

sum bloodglucose_ord diabetesdummy_mf_both bmi age female_new education occupt married bankaccnt last_ate last_drink smokecigrtt smokepipe chewtobacco snuff smokecigar paangutkha paanwithtobacco drinkalcohol fried1 aerated1 wealth_new religion sc st obc insurance bpl familystruc hh_members region

// S3 Table

**Average Marginal Effects of BMI on Self-Reported Diabetes Status: Probit Model Estimates based on Full Sample Data**

probit diabetesdummy_mf_both bmi age i.female bankaccnt i.wealth i.religion sc st obc insurance bpl i.familystruc i.education hh_members region smokecigrtt smokepipe chewtobacco snuff smokecigar paangutkha paanwithtobacco drinkalcohol fried1 aerated1 i.statecode , vce(robust)

margins , dydx( bmi ) over ( overweigh_obese ) pwcompare (group)

margins , dydx( bmi ) over ( overweigh_obese ) pwcompare

margins , dydx( bmi ) over ( overweigh_obese23 ) pwcompare (group)

margins , dydx( bmi ) over ( overweigh_obese23 ) pwcompare

// S4 Table

**Average Marginal Effects of BMI on Self-Reported Diabetes Status: Probit and IV-Probit Model Estimates for Married Couples Sub-Sample**

**Porbit Model**

**Columns 1, 2 and 3**

probit diabetesdummy_mf_both bmi_head age i.female bankaccnt i.wealth i.religion sc st obc insurance bpl i.familystruc i.education hh_members region smokecigrtt smokepipe chewtobacco snuff smokecigar paangutkha paanwithtobacco drinkalcohol fried1 aerated1 i.statecode if dumbmihwpositive==1 , vce(robust)

margins, dydx ( bmi_head ) pred(pr) over ( female_new)

margins, dydx ( bmi_head ) pred(pr) over ( female_new) pwcompare (group)

margins, dydx ( bmi_head ) pred(pr) over ( female_new) pwcompare

margins, dydx ( bmi_head ) pred(pr) over ( region)

margins, dydx ( bmi_head ) pred(pr) over ( region) pwcompare (group)

margins, dydx ( bmi_head ) pred(pr) over ( region) pwcompare

margins, dydx ( bmi_head ) pred(pr) over ( wealth)

margins, dydx ( bmi_head ) pred(pr) over ( wealth) pwcompare (group)

margins, dydx ( bmi_head ) pred(pr) over ( wealth) pwcompare

**IV-Porbit Model**

**Columns 4, 5 and 6 **

eststo RDjune5: ivprobit diabetesdummy_mf_both age i.female bankaccnt i.wealth i.religion sc st obc insurance bpl i.familystruc i.education hh_members region smokecigrtt smokepipe chewtobacco snuff smokecigar paangutkha paanwithtobacco drinkalcohol fried1 aerated1 i.statecode (bmi_head = bmi_wife_husb) , vce(cluster statecode)

margins, dydx ( bmi_head ) pred(pr) over ( female_new)

margins, dydx ( bmi_head ) pred(pr) over ( female_new) pwcompare (group)

margins, dydx ( bmi_head ) pred(pr) over ( female_new) pwcompare

margins, dydx ( bmi_head ) pred(pr) over ( region)

margins, dydx ( bmi_head ) pred(pr) over ( region) pwcompare (group)

margins, dydx ( bmi_head ) pred(pr) over ( region) pwcompare

margins, dydx ( bmi_head ) pred(pr) over ( wealth)

margins, dydx ( bmi_head ) pred(pr) over ( wealth) pwcompare (group)

margins, dydx ( bmi_head ) pred(pr) over ( wealth) pwcompare

// S5 Table

**Average Marginal Effects of BMI on Ordinal Blood Glucose Levels: Ordered Probit Model Estimates based on Full Sample Data**

oprobit bloodglucose_ord bmi age i.female bankaccnt i.wealth i.religion sc st obc insurance bpl i.familystruc i.education married hh_members region last_ate last_drink smokecigrtt smokepipe chewtobacco snuff smokecigar paangutkha paanwithtobacco drinkalcohol fried1 aerated1 i.statecode, vce(robust)

margins , dydx( bmi ) over (female) pwcompare (group)

margins , dydx( bmi ) over (female) pwcompare

margins , dydx( bmi ) over (region) pwcompare (group)

margins , dydx( bmi ) over (region) pwcompare

margins , dydx( bmi ) over (wealth) pwcompare (group)

margins , dydx( bmi ) over (wealth) pwcompare
