# Supplementary material for: Does a rise in BMI cause an increased risk of diabetes?: Evidence from India
Source: PLoS One. 2020 Apr 1;15(4):e0229716. doi: 10.1371/journal.pone.0229716 (PMC7112218; doi:10.1371/journal.pone.0229716)
Supplement: S1 Fig — Source: Figure constructed by author based on NFHS data for year 2015–16. Blood glucose levels are measured in mg/dl. (DOCX) [file pone.0229716.s002.docx]

**S1 Figure: BMI Distribution by Blood Glucose Levels**

**
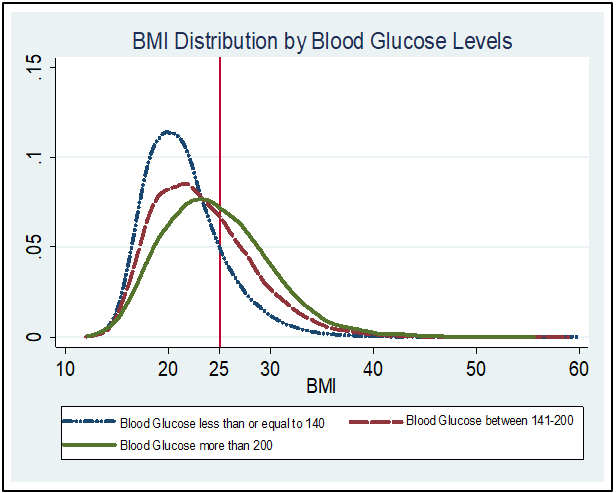
**

Source: Figure constructed by author based on NFHS data for year 2015-16.

Note: Blood glucose levels are measured in mg/dl.
