# Supplementary material for: Does a rise in BMI cause an increased risk of diabetes?: Evidence from India
Source: PLoS One. 2020 Apr 1;15(4):e0229716. doi: 10.1371/journal.pone.0229716 (PMC7112218; doi:10.1371/journal.pone.0229716)
Supplement: S1 Table — (DOCX) [file pone.0229716.s003.docx]

**S1 Table: List of Variables with Definition and Type**

| **Variable** | **Definition** | **Type** |
| --- | --- | --- |
| **Health Outcome Variables:** | | |
| Ordinal Blood Glucose Levels | - BG = 0 if blood glucose is less than or equal to140 mg/dl - BG = 1 if between 141 and 200 mg/dl - BG = 2 if higher than 200 mg/dl | Ordinal |
| Self-Reported Diabetes Status | - D = 0 if non-diabetic - D = 1 if diabetic | Binary |
| **List of Independent Variables:** | | |
| **Individual Characteristics:** | | |
| Body Mass Index | Person’s weight is kilograms divided by square of his/her height in meters (kg/m^2^). | Continuous |
| Age | Age in years. | Continuous |
| Gender | - = 0 if Male^@^ - = 1 if Female | Binary |
| Education | - = 0 if no education or preschool^@^ - = 1 if Primary - = 2 if Secondary - = 3 if Higher | Ordinal |
| Marital Status | - = 0 if Never married^@^ ^[[1]](#footnote-1)^ - = 1 if Married | Binary |
| Bank Account | - = 0 if individual does not have bank account^@^ - = 1 if individual has bank account | Binary |
| Time since last ate | Time since last ate (in hours). Time is recorded before blood glucose measurements are taken. | Continuous |
| Time since last drink | Time since last drink (in hours), something other than plain water. Time is recorded before blood glucose measurements are taken. | Continuous |
| Behavioural Risk Factors^[[2]](#footnote-2)^ | - = 1 if smokes cigarette, 0 otherwise^@^ - = 1 if smokes pipe, 0 otherwise^@^ - = 1 if chews tobacco, 0 otherwise^@^ - = 1 if snuffs, 0 otherwise^@^ - = 1 if smokes cigar, 0 otherwise^@^ - = 1 if chews paan or gutkha, 0 otherwise^@^ - = 1 if chews paan with tobacco, 0 otherwise^@^ - = 1 if drinks alcohol, 0 otherwise^@^ | Binary |
| Eating Habits^[[3]](#footnote-3)^ | - = 1 if eats fried food daily or weekly, 0 otherwise^@^ - = 1 if drinks aerated drink daily or weekly, 0 otherwise^@^ | Binary |
| **S1 Table (Continued)** | | |
| **Variable** | **Definition** | **Type** |
| **Household Characteristics:** | | |
| Wealth Quintile | - = 0 if poorest^@^ - = 1 if poorer - = 2 if middle - = 3 if richer - = 4 if richest | Ordinal |
| Religion | - = 0 if Hindu^@^ - = 1 if Muslim - = 2 if Christian - = 3 if Sikh - = 4 if Buddhist/neo-Buddhist - = 5 if Jain - = 6 if Jewish - = 7 if Parsi/Zoroastrian - = 8 if no religion - = 9 if some other religion | Ordinal |
| Caste^[[4]](#footnote-4)^ | - = 1 if Scheduled Caste, 0 otherwise^@^ - = 1 if Scheduled Tribe, 0 otherwise^@^ - = 1 if Other Backward Classes, 0 otherwise^@^ | Binary |
| Insurance | - = 0 if any usual member of household is not covered by a health scheme or health insurance^@^ - = 1 if any usual member of household is covered by a health scheme or health insurance | Binary |
| Below Poverty Line | - = 0 if household does not have BPL card^@^ - = 1 if household has BPL card | Binary |
| Family Structure | - = 0 if nuclear family^@^ - = 1 if non-nuclear or joint family | Binary |
| Number of Household Members | Number of total household members in all age groups. | Continuous |
| Region | - = 0 if Rural^@^ - = 1 if Urban | Binary |

^@^ Indicates the base category.

1. Includes married but gauna not done. [↑](#footnote-ref-1)
2. Contains a set of eight dummy variables. [↑](#footnote-ref-2)
3. Contains a set of two dummy variables. [↑](#footnote-ref-3)
4. Contains a set of three dummy variables. [↑](#footnote-ref-4)
