# Supplementary material for: Does a rise in BMI cause an increased risk of diabetes?: Evidence from India
Source: PLoS One. 2020 Apr 1;15(4):e0229716. doi: 10.1371/journal.pone.0229716 (PMC7112218; doi:10.1371/journal.pone.0229716)
Supplement: S2 Table — (DOCX) [file pone.0229716.s004.docx]

**S2 Table: Descriptive Statistics**

| **Variable** | **Observations** | **Mean** | **Standard Deviation** | **Minimum Value** | **Maximum Value** |
| --- | --- | --- | --- | --- | --- |
| **Individual Characteristics** | | | | | |
| Self-Reported Diabetes Status | 784042 | 0.015 | 0.120 | 0 | 1 |
| Ordinal Blood Glucose levels | 806905 | 0.070 | 0.294 | 0 | 2 |
| Blood Glucose levels – Actual Values (in mg/dl) | 806905 | 104.689 | 29.602 | 20 | 499 |
| Body Mass Index (in kg/m^2^) | 811465 | 21.714 | 4.094 | 12.01 | 59.96 |
| Age (in years) | 811465 | 30.066 | 9.967 | 15 | 54 |
| Gender | 811465 | 0.863 | 0.344 | 0 | 1 |
| Education | 809904 | 1.483 | 0.994 | 0 | 3 |
| Married | 782387 | 0.732 | 0.443 | 0 | 1 |
| Bank Account | 810731 | 0.914 | 0.281 | 0 | 1 |
| Time since last ate (in hours) | 805589 | 3.132 | 3.543 | 0 | 48 |
| Time since last drink (in hours) | 800779 | 5.384 | 14.049 | 0 | 95 |
| **Behavioural Risk Factors** | | | | | |
| Smokes Cigarette | 795856 | 0.024 | 0.154 | 0 | 1 |
| Smokes Pipe | 795856 | 0.001 | 0.025 | 0 | 1 |
| Chews Tobacco | 795856 | 0.012 | 0.108 | 0 | 1 |
| Snuffs | 795856 | 0.001 | 0.034 | 0 | 1 |
| Smokes Cigar | 795856 | 0.001 | 0.037 | 0 | 1 |
| Chews Paan or Gutkha | 795856 | 0.049 | 0.216 | 0 | 1 |
| Chews Paan with Tobacco | 795856 | 0.043 | 0.204 | 0 | 1 |
| Consumes Alcohol | 795856 | 0.065 | 0.246 | 0 | 1 |
| **Eating Habits** | | | | | |
| Fried Food | 795856 | 0.455 | 0.498 | 0 | 1 |
| Aerated Drinks | 795856 | 0.242 | 0.429 | 0 | 1 |
| **Household Characteristics** | | | | | |
| Wealth Quintile | 811465 | 1.983 | 1.384 | 0 | 4 |
| Religion | 811465 | 0.520 | 1.26 | 0 | 9 |
| SC | 811465 | 0.181 | 0.385 | 0 | 1 |
| ST | 811465 | 0.182 | 0.386 | 0 | 1 |
| OBC | 811465 | 0.387 | 0.487 | 0 | 1 |
| Insurance | 806832 | 0.262 | 0.440 | 0 | 1 |
| Below Poverty Line | 810055 | 0.386 | 0.487 | 0 | 1 |
| Family Structure | 811465 | 0.503 | 0.500 | 0 | 1 |
| Number of Household Members | 811465 | 5.772 | 2.651 | 1 | 41 |
| Region | 811465 | 0.292 | 0.455 | 0 | 1 |

Note: Values are based on full sample.
