# Supplementary material for: Does a rise in BMI cause an increased risk of diabetes?: Evidence from India
Source: PLoS One. 2020 Apr 1;15(4):e0229716. doi: 10.1371/journal.pone.0229716 (PMC7112218; doi:10.1371/journal.pone.0229716)
Supplement: S3 Table — (DOCX) [file pone.0229716.s005.docx]

**S3 Table: Average Marginal Effects of BMI on Self-Reported Diabetes Status: Probit Model Estimates based on Full Sample Data**

|  |  | **Probit Model** | |
| --- | --- | --- | --- |
| **Marginal Effects** |  | **WHO International BMI Classification** | **WHO Asian BMI Classification** |
|  |  | **(1)** | **(2)** |
| **Overweight or Obese Individuals** |  | 0.0023***  (0.00008) | 0.0019***  (0.00006) |
| **Non-Overweight Individuals** |  | 0.0008***  (0.00002) | 0.0007***  (0.00002) |
| **Difference^#^** |  | 0.0015***  (0.00006) | 0.0013***  (0.00005) |
| **Controls** |  | Yes | |
| **State Fixed Effects** |  | Yes | |
| **Observations** |  | 776394 | |
| **Wald chi2** |  | 10987.47 | |
| **P-Value** |  | 0.0000 | |
| **Pseudo R^2^** |  | 0.1256 | |

*** represents significance at 1% significance level.

Delta-Method standard errors are reported in parentheses.

^#^ Difference is ME(Overweight and Obese) – ME(Non-Overweight). And dof = degrees of freedom.

Controls include individual and household characteristics, behavioural risk factors and eating habits.

Individual and household characteristics include age, gender, education, marital status, bank account, household characteristics such as wealth quintile, religion, caste, insurance, below poverty line, family structure, number of household members and region.

Behavioural risk factors include smoking cigarette, smoking pipe, chewing tobacco, snuffing, smoking cigar, chewing paan or gutkha, chewing paan with tobacco and drinking alcohol.

Eating habits include daily or weekly consumption of fried foods and aerated drinks.
