# Supplementary material for: Does a rise in BMI cause an increased risk of diabetes?: Evidence from India
Source: PLoS One. 2020 Apr 1;15(4):e0229716. doi: 10.1371/journal.pone.0229716 (PMC7112218; doi:10.1371/journal.pone.0229716)
Supplement: S4 Table — (DOCX) [file pone.0229716.s006.docx]

**S4 Table: Average Marginal Effects of BMI on Self-Reported Diabetes Status: Probit and IV-Probit Model Estimates for Married Couples Sub-Sample**

|  | |  | **Probit Model** | | |  | **IV-Probit Model** | | |
| --- | --- | --- | --- | --- | --- | --- | --- | --- | --- |
|  | |  | **Gender** | **Region** | **Wealth Quintile** |  | **Gender** | **Region** | **Wealth Quintile** |
|  | |  | **(1)** | **(2)** | **(3)** |  | **(4)** | **(5)** | **(6)** |
|  | |  | **Male** | **Urban** | **Richest** |  | **Male** | **Urban** | **Richest** |
| **Marginal Effects** | |  | 0.0020***  (0.0002) | 0.0026***  (0.0003) | 0.0035***  (0.0004) |  | 0.0069***  (0.0015) | 0.0089***  (0.0019) | 0.0116***  (0.0024) |
|  | |  | **Female** | **Rural** | **Poorest** |  | **Female** | **Rural** | **Poorest** |
| **Marginal Effects** | |  | 0.0012***  (0.0009) | 0.0017***  (0.0002) | 0.0010***  (0.0001) |  | 0.0048*  (0.0098) | 0.0059***  (0.0013) | 0.0038***  (0.0010) |
|  | |  | **Difference^#^** | **Difference^#^** | **Difference^#^** |  | **Difference^#^** | **Difference^#^** | **Difference^#^** |
|  | |  | 0.0007*  (0.0004) | 0.0009***  (0.0001) | 0.0025***  (0.0003) |  | 0.0020  (0.0020) | 0.0030***  (0.0007) | 0.0078***  (0.0015) |
| **Controls** |  | | Yes | | |  | Yes | | |
| **State Fixed Effects** |  | | Yes | | |  | Yes | | |
| **Observations** | |  | 43202 | | |  | 43202 | | |
| **Wald chi2** | |  | 1010.93 | | |  | 234600.29 | | |
| **P-Value** | |  | 0.0000 | | |  | 0.0000 | | |
| **Pseudo R^2^** | |  | 0.1072 | | |  |  | | |
| **Wald test of exogeneity, chi2** | |  |  | | |  | 18.73 | | |
| **P-Value** | |  |  | | |  | 0.0000 | | |

***, ** and * represents significance at 1%, 5% and 10% significance level.

Delta-Method standard errors are reported in parentheses.

^#^ (1) Difference is ME(Male) – ME(Female); (2) Difference is ME(Urban) – ME(Rural) and (3) Difference is ME(Richest) – ME(Poorest).

Note: Probit and IV-Probit models do not include marital status as a control. Marital status is omitted in the restricted sample as the sample comprises of only married individuals.

Controls include individual and household characteristics, behavioural risk factors and eating habits.

Individual and household characteristics include age, gender, education, bank account, household characteristics such as wealth quintile, religion, caste, insurance, below poverty line, family structure, number of household members and region.

Behavioural risk factors include smoking cigarette, smoking pipe, chewing tobacco, snuffing, smoking cigar, chewing paan or gutkha, chewing paan with tobacco and drinking alcohol.

Eating habits include daily or weekly consumption of fried foods and aerated drinks.
