# Supplementary material for: Does a rise in BMI cause an increased risk of diabetes?: Evidence from India
Source: PLoS One. 2020 Apr 1;15(4):e0229716. doi: 10.1371/journal.pone.0229716 (PMC7112218; doi:10.1371/journal.pone.0229716)
Supplement: S5 Table — (DOCX) [file pone.0229716.s007.docx]

**S5 Table: Average Marginal Effects of BMI on Ordinal Blood Glucose Levels: Ordered Probit Model Estimates based on Full Sample Data**

|  | | **Ordered Probit Model** | | | | |
| --- | --- | --- | --- | --- | --- | --- |
| **Marginal Effects** |  | **Gender** | | | | |
|  |  | **Male** |  | **Female** |  | **Difference^#^** |
| **Normal Blood Glucose (Blood Glucose ≤ 140)** |  | -0.0054***  (0.0001) |  | -0.0038***  (0.0001) |  | -0.0015***  (0.00005) |
| **Prediabetes (141 ≤ Blood Glucose ≤ 200)** |  | 0.0039***  (0.0001) |  | 0.0030***  (0.00005) |  | 0.0009***  (0.00003) |
| **Diabetes (Blood Glucose > 200)** |  | 0.0014***  (0.00003) |  | 0.0009***  (0.00002) |  | 0.0006***  (0.00002) |
|  |  | **Region** | | | | |
|  |  | **Urban** |  | **Rural** |  | **Difference^#^** |
| **Normal Blood Glucose (Blood Glucose ≤ 140)** |  | -0.0047***  (0.00008) |  | -0.0038***  (0.00006) |  | -0.0009***  (0.00003) |
| **Prediabetes (141 ≤ Blood Glucose ≤ 200)** |  | 0.0035***  (0.00006) |  | 0.0030***  (0.00005) |  | 0.0005***  (0.00002) |
| **Diabetes (Blood Glucose > 200)** |  | 0.0012***  (0.00002) |  | 0.0009***  (0.00002) |  | 0.0003***  (0.00001) |
|  |  | **Wealth Quintile** | | | | |
|  |  | **Richest** |  | **Poorest** |  | **Difference^#^** |
| **Normal Blood Glucose (Blood Glucose ≤ 140)** |  | -0.0049***  (0.00008) |  | -0.0033***  (0.00006) |  | -0.0016***  (0.00005) |
| **Prediabetes (141 ≤ Blood Glucose ≤ 200)** |  | 0.0036***  (0.00006) |  | 0.0026***  (0.00005) |  | 0.0010***  (0.00003) |
| **Diabetes (Blood Glucose > 200)** |  | 0.0013***  (0.00003) |  | 0.0007***  (0.00002) |  | 0.0060***  (0.00002) |
| **Controls** | | Yes | | | | |
| **State Fixed Effects** | | Yes | | | | |
| **Observations** |  | 748,995 | | | | |
| **Wald chi2** |  | 26968.90 | | | | |
| **P-Value** |  | 0.0000 | | | | |
| **Pseudo R^2^** |  | 0.0901 | | | | |

*** represents significance at 1% significance level.

Delta-Method standard errors are reported in parentheses.

^#^ (1) Difference is ME(Male) – ME(Female); (2) Difference is ME(Urban) – ME(Rural) and (3) Difference is ME(Richest) – ME(Poorest).

Controls include individual and household characteristics, behavioural risk factors and eating habits.

Individual and household characteristics include age, gender, education, marital status, bank account, household characteristics such as wealth quintile, religion, caste, insurance, below poverty line, family structure, number of household members, region and time since last ate and drank.

Behavioural risk factors include smoking cigarette, smoking pipe, chewing tobacco, snuffing, smoking cigar, chewing paan or gutkha, chewing paan with tobacco and drinking alcohol.

Eating habits include daily or weekly consumption of fried foods and aerated drinks.
